# Optimization Preparation and Evaluation of Chitosan Grafted Norfloxacin as a Hemostatic Sponge

**DOI:** 10.3390/polym15030672

**Published:** 2023-01-28

**Authors:** Yu Cheng, Qian Yang, Jiyuan Wang, Zhang Hu, Chengpeng Li, Saiyi Zhong, Na Huang

**Affiliations:** 1 Faculty of Chemistry and Environment Science, Guangdong Ocean University, Zhanjiang 524088, China; 2 College of Food Science and Technology, Guangdong Ocean University, Zhanjiang 524088, China

**Keywords:** chitosan, norfloxacin, optimization, anti-bacteria, hemostasis

## Abstract

Considering the great harm to the human body caused by severe and massive bleeding, in this study, chitosan-grafted norfloxacin (CTS-NF) composites were prepared with chitosan (CTS) and norfloxacin (NF) as raw materials by a 1-ethyl-3-(3-dimethylaminopropyl) carbodiimide-mediated coupling method to solve the limitations of slow hemostatic and poor anti-infective effects of current dressings on the market. The effects of the mass ratio of CTS to NF (M_CTS_/M_NF_), reaction temperature T and reaction time t on the grafting rate (η%) of the products were investigated through single factor tests. The preparation process was optimized with the η% as an evaluation index by means of the Box–Behnken test design and response surface analysis. The antimicrobial activity was evaluated by inhibition zone assay, and the hemostatic activity of the prepared composites was evaluated in vitro and in vivo. The results suggested that the optimum preparation conditions were the mass ratio of CTS to NF (M_CTS_/M_NF_) 5:3, reaction temperature 65 °C, and reaction time 4 h. Under this condition, the η% of CTS-NF was 45.5%. The CTS-NF composites displayed significant antimicrobial activities. Moreover, in vitro hemostasis results revealed that the CTS-NF composite had a lower blood clotting index and absorbed red blood cells to promote aggregation. In vivo ear and live hemostasis, the CTS-NF groups showed short hemostatic time (49.75 ± 3.32 s and 50.00 ± 7.21 s) and more blood loss (0.07 ± 0.010 g and 0.075 ± 0.013 g). The results showed that CTS-NF reduced the bleeding time and volume, exhibiting a significant coagulation effect. Therefore, the CTS-NF sponge is expected to be a new, effective hemostatic and antibacterial material in the future.

## 1. Introduction

Bleeding is one of the most critical issues in war, emergency accidents and surgical operations. Wound infection is one of the reasons for slow hemostasis [1]. The hemostatic and antibacterial dressings are more efficient in hemostasis and bacteriostasis [2,3], which allows wounds to heal more rapidly and thus relieve the patient’s pain. Traditional gauze dressings can no longer meet modern wound healing requirements. It is extremely urgent to develop an ideal combined hemostatic and antibacterial dressing based on the medical needs. 

Chitosan (CTS), a deacetylated product of chitin, is a polymeric biomaterial of wide origin. In addition to excellent biocompatibility, biodegradability, antibacterial properties, and adsorption and film-forming properties [4,5], CTS also exhibits a significant hemostatic effect [6,7]. Rapid hemostatic agents such as HemCon Bandage and Celox, which use CTS as the primary hemostatic material, are applied in clinical practice [8]. 

Fluoroquinolone antibacterial agents have been effectively used in hemostatic materials [9,10]. It was found that the addition of NF significantly improved the hemostatic process by inhibiting bacterial infection [11,12]. As a fluoroquinolone broad-spectrum antibacterial agent [13], norfloxacin (NF), which can effectively inhibit gram-negative and gram-positive bacteria and has antibacterial activity against *Bacillus dysenteriae* and *Escherichia coli* (*E. coli*) [14,15], among others, can be used as a bacteriostatic agent during wound treatment [16,17]. However, because NF is degraded rapidly in vivo and has poor bioavailability [18,19], its bacteriostatic effect cannot be fully exerted if directly applied to the hemostasis process alone because of its limited permeation rate resulting from low biocompatibility, thereby greatly reducing the bacteriostatic effect of NF in the hemostasis process.

The bacteriostatic effect of single CTS hemostatic material is significantly insufficient in the process of hemostasis [20,21,22], which reduces the hemostatic effect of CTS hemostatic material, while NF is less biocompatible. In previous studies, chitosan and norfloxacin were physically added or hydrogen bonded, which were not stable enough. To solve the above limitations, a new hemostatic and antibacterial composite material was prepared by grafting chitosan, which has good biocompatibility, and NF, which has a significant antibacterial effect under aqueous phase conditions, using *1-ethyl-3-(3-dimethylaminopropyl) carbodiimide* (EDC) as catalysts via chemical modification [23,24]. Its hemostatic effect was evaluated by whole blood clotting index (BCI) measurement and coagulation in vitro and in vivo, so as to provide a theoretical basis for the application of CTS-NF composites in the pharmaceutical field in the future.

## 2. Materials and Methods

### 2.1. Materials 

High-density CTS (Mw 100 kDa, degree of deacetylation ≥ 95%, density ≥ 0.6 g/mL) and NF were both analytical grade (purity ≥ 98%) and purchased from Shanghai Macklin Biochemical Co., Ltd. (Shanghai, China). EDC was purchased from Shanghai Yuanye Bio-Technology Co., Ltd. ((purity ≥ 99%), Shanghai, China). The prothrombin time (PT) (lyophilized type), activated partial thrombin (APTT) (injected flower acid), thrombin time (TT) (lyophilized type) and fibrinogen (FIB) (lyophilized) assay kits were purchased from Shanghai Sun Biotechnology Co., Ltd. (Shanghai, China), SEM(S-4800) was purchased from Hitachi, Japan. Microplate reader (DNM-9602) was purchased from Beijing Planck New Technology Co., Ltd., China.

### 2.2. Preparation of CTS-NF

**Figure 1:**
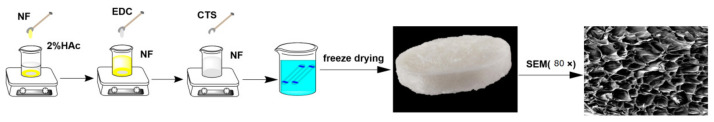

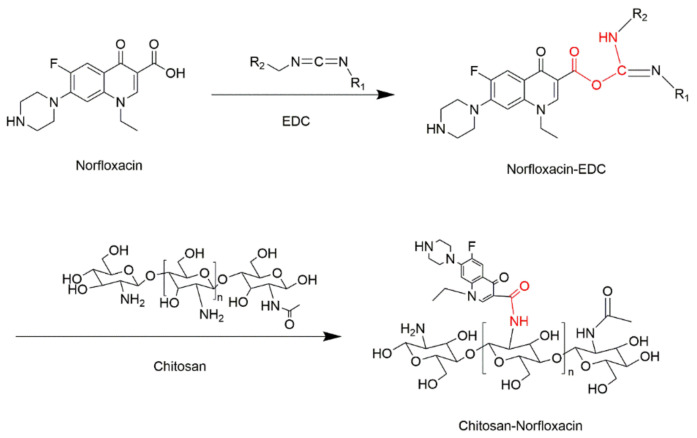
Preparation of the CTS-NF composite sponge.

The CTS-NF composite sponges were prepared with chitosan (CTS) and norfloxacin in a similar way to our previous work [7]. NF was dissolved in 2% acetic acid solution and the crosslinker EDC was added and activated by stirring at room temperature. In addition, a given amount of CTS was dissolved in 2% acetic acid solution and the resulting solution was added slowly into the above reaction system. The mixed solution was continuously stirred for a given period of time at a specific temperature. The reaction product was dialyzed against purified water and freeze-dried to obtain CTS-NF composite sponge (Figure 1).

### 2.3. Morphology Observation of CTS-NF

The appearance of the sponges was recorded using a DSC-TX10 digital camera and the micromorphology was observed after the gold sputtering treated by scanning electron microscopy (SEM).

### 2.4. X-ray Diffraction (XRD) Characterization

The XRD patterns of the samples were measured by an X-ray diffractometer with Kα radiation (wavelength = 0.15405 nm), a voltage of 40 kV and a current of 45 mA, the 2θ range of 10–80° and a scanning speed of 10°/min.

### 2.5. Measurement of the Grafting Ratio of CTS-NF

**Figure 2:**
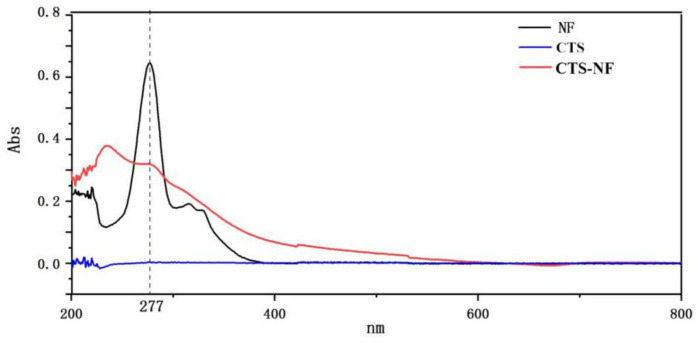
UV–Vis spectra of CTS, NF, and CTS-NF.

The grafting ratio of CTS-NF (η%) was determined using a method in the literature [25]. The UV-Vis spectra of chitosan, norfloxacin, and CTS-NF are presented in Figure 2. NF solutions at the concentrations of 1.0, 2.0, 3.0, 4.0, 5.0 and 6.0 μg/mL were prepared and their absorbance was determined at 277 nm, and the calibration curve formula (1) of NF was obtained using the concentration of NF as the abscissa. After obtaining the sample concentration, the η% of the composite was calculated based on Formula (2).

(1) $$A=0.11261c+0.00232,R^{2}=0.99903$$

(2) $$\eta \%=\frac{\mathrm{CVM}}{m}\times 100\%\;$$

where A is the UV absorbance, C is the NF concentration, V is the volume of NF, M is the NF molar mass, m is the mass of sponge, and η(%) is the grafting ratio. 

### 2.6. Single-Factor Tests

Three factors, the mass ratio of CTS to NF (M_CTS_/M_NF_), reaction temperature (T) and reaction time (t), were selected for single factor tests and M_CTS_/M_NF_ of 5:1, 5:2, 5:3, 5:4, and 5:5, T of 50, 55, 60, 65, and 70 °C, and t of 2, 3, 4, 5, and 6 h, respectively, were adopted for single factor tests. When one factor was examined, the other factors were fixed as follows: M_CTS_/M_NF_ 5:3, reaction temperature 65 °C, and reaction time 4 h. To obtain the grafting conjugate with the maximum η%, response surface methodology was used to optimize the reaction process based on the results of the single-factor tests.

### 2.7. Response Surface Methodology Optimization

On the basis of the results of the single-factor tests, DesignExpert response surface software was applied for analysis using the η% as the response value to explore the three factors of M_CTS_/M_NF_, T and t to optimize and prepare CTS-NF composites of different η%.

### 2.8. In Vitro Antimicrobial Activity Evaluation by MIC Assay

The MIC values were determined using a broth dilution method [26] against gram-negative E. coli ATCC 25922 and gram-positive S. aureus ATCC 29213. The starting concentrations of tested compounds were 64 mg/mL. The solution of compound 150 μL was added to 150 μL of bacterial culture (106 CFU/mL) at the first well of flat bottomed 96-well tissue culture plates. The solution was then double diluted. Bacterial culture solution containing appropriate compound (150 μL) was discarded at the last well in order to ensure 150 μL volume of bacterial culture in every well. The plate was incubated at 37 °C overnight in electro-heating standing-temperature cultivator before the measurement of the absorbance value. The optical density values at 600 nm were measured using a multifunction microplate reader.

### 2.9. Whole Blood Clotting and Red Blood Cell Aggregation Assays

Fresh rabbit blood was drawn using a blood collection tube containing a sodium citrate anticoagulant and set aside. A sample of 10 mg was added into a beaker and incubated at 37 °C for 5 min, to which was subsequently added 200 μL fresh anticoagulated rabbit blood followed by 20 μL 0.2 M CaCl_2_ solution and incubated at 37 °C for 5 min. Subsequently, 25 mL deionized water was added and shaken at 37 °C in a constant-temperature shaker. The supernatant was filtered, and the absorbance of the supernatant obtained by UV scanning at 545 nm. In addition, a medical gauze group and a blank control group were established, and each group was subject to three tests and the mean value was taken. The BCI [27] was obtained using the following Formula (3):

(3) $$\mathrm{BCI}={\mathrm{Abs}}_{\mathrm{sample}}/{\mathrm{Abs}}_{\mathrm{blank}}\times 100\%\;$$

where Abs of sample and Abs of blank refer to the absorbance of the solution with and without the sample, respectively.

For aggregation of red blood cells, these cells were collected by centrifugation of fresh anticoagulated at 3000 rpm for 15 min and diluted 10 times in phosphate buffered saline (PBS). The sponge of 10 mg was transferred into a tube and incubated at 37 °C for 5 min, to which was subsequently added 200 μL red blood cell solution, and incubated at 37 °C for 0.5 h. The red blood cell solution on the surface of the sample was removed, and the sample was washed with a PBS solution to remove free red blood cells. Subsequently, the samples were treated with 2.5% *v*/*v* glutaraldehyde solution for 0.5 h to fix the adherent red blood cells and washed with ethanol solution in a gradient concentration between 70 and 100%. The aggregation of red blood cells in the freeze dried samples was observed by SEM.

### 2.10. Blood Plasma Coagulation Assay In Vitro

Fresh anticoagulated blood was centrifuged at 3000 rpm for 15 min, and the platelet-poor plasma was collected for plasma coagulation analysis. The APTT, PT, TT, and FIB were determined using a semi-automatic coagulation analyzer (HF6000-4, Jinan Hanfang Medical Instrument Co., Ltd.) according to the respective kit instructions and physiological saline was used as blank control. 

### 2.11. Animal Hemostasis Experiments

Twenty New Zealand rabbits, half female and half male, were purchased from Guangdong Medical Laboratory Animal Center (Foshan, China). All rabbits were randomly divided into 2 groups, including CTS-NF and medical gauzegroup (as control). All animal experiments were approved by the Animal Care and Use Committee of Guangdong Ocean University, China (SYXK20180147).

#### 2.11.1. Hemorrhage Model of Ear Artery

The rabbits were anesthetized with 3% sodium pentobarbital (30 mg/kg) and the ears were dehaired. The ear artery and vein were cut horizontally. When the blood from the wound covered the wound surface, a hemostatic agent was immediately placed on the bleeding site with sterile medical gauze, followed by manual compression to stop the bleeding. The bleeding was observed every 30 s until it stopped, and the hemostatic time was recorded.

#### 2.11.2. Liver Hemorrhage Model

The ear hemorrhage experiment did not have any severe effects on important organs. Thus, after the ear wounds had recovered, 20 rabbits were used to establish a hepatic hemorrhage model. After anesthesia, the abdomen was cut open with a surgical knife and the liver was carefully pulled out. A“+” wound of 1 cm (length) × 1 cm (width) × 0.3 cm (depth) was made in the hepatic lobe. The bleeding was stopped by manual compression as described in Section 2.10. The hemostatic samples were weighed and the blood loss was calculated.

### 2.12. Statistical Analysis

Data were processed with an IBM SPSS Statistics v22 and analyzed using independent sample t tests. Numerical data are expressed as the means ± standard deviations (SDs), and the differences among groups were analyzed using a one-way analysis of variance (ANOVA). Here, *p* < 0.05 and *p* < 0.01 indicate statistically significant and highly significant differences, respectively.

## 3. Results and Discussion

### 3.1. Characterization of CTS-NF

**Figure 3:**
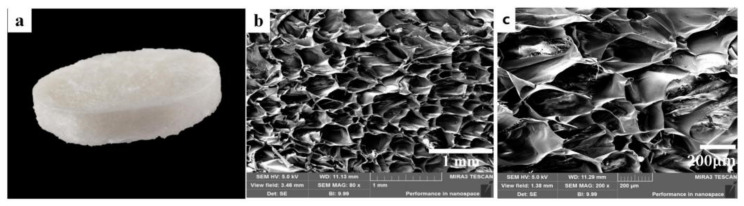
Morphology of the CTS-NF sponge (**a**) SEM micrograph of the cross-section (80×) (**b**). the internal section (200×) (**c**).

As shown by the image of the CTS-NF sponge photographed with a digital camera (Figure 3a), the sample overall is a white sponge with a relatively flat and elastic surface and many obvious small holes. The porous fluffy structure with a certain thickness allows the sample to have a good adsorption of blood as well as sufficient space to contain the blood at the wound, which contributes to accelerating hemostasis. As shown in the image of the CTS-NF sponge under the electron microscope at 80× (Figure 3b) and at 200× (Figure 3c), the pore structure of the composite sponge can be clearly observed and the pores are arranged regularly and densely.

### 3.2. XRD Analysis

**Figure 4:**
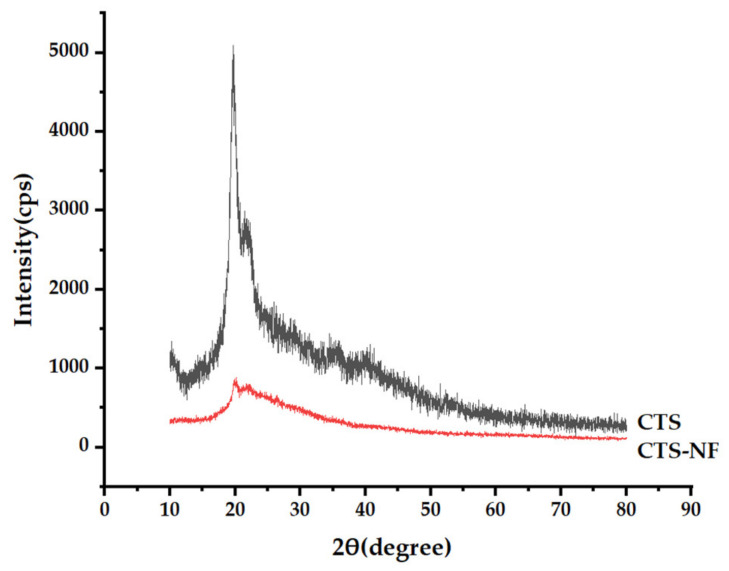
XRD patterns of CTS-NF and CTS.

As illustrated in Figure 4, the XRD curve of CTS indicated typical peaks at 2θ values of 20.1°. Chitosan has obvious crystal reflection characteristics. As shown in the CTS-NF spectrum, when imine bonding was formed between CTS and NF, the crystalline peaks belonging to CTS disappeared, indicating that the crystallinity of CTS was better than that of CTS-NF.

### 3.3. Single-Factor Analysis

#### 3.3.1. Effect of the Mass Ratio M_CTS_/M_NF_ on the Grafting Rate η%

**Figure 5:**
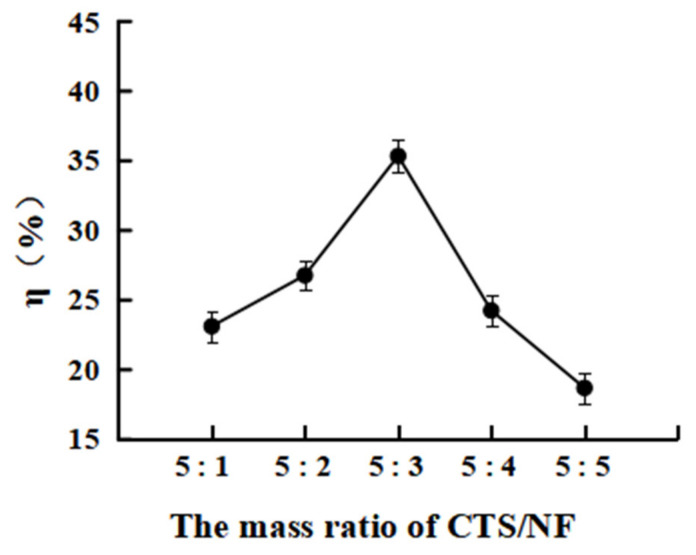
Effect of the mass ratio of M_CTS_/M_NF_ on the grafting rate.

As shown in Figure 5, the η% increased from 23.1 ± 1.08 to 35.4 ± 1.34 as the M_CTS_/M_NF_ was raised from 5:1 to 5:3. However, the η% gradually decreased as the M_CTS_/M_NF_ was further increased, which was probably because the grafting reaction gradually reached a dynamic equilibrium as the ratio of NF increased. However, the combined effects of electrostatic repulsion and steric hindrance of NF and CTS inhibited the reaction rather than the mass ratio of NF as this was further enhanced [28].

#### 3.3.2. Effect of the Reaction Time on the Grafting Rate

**Figure 6:**
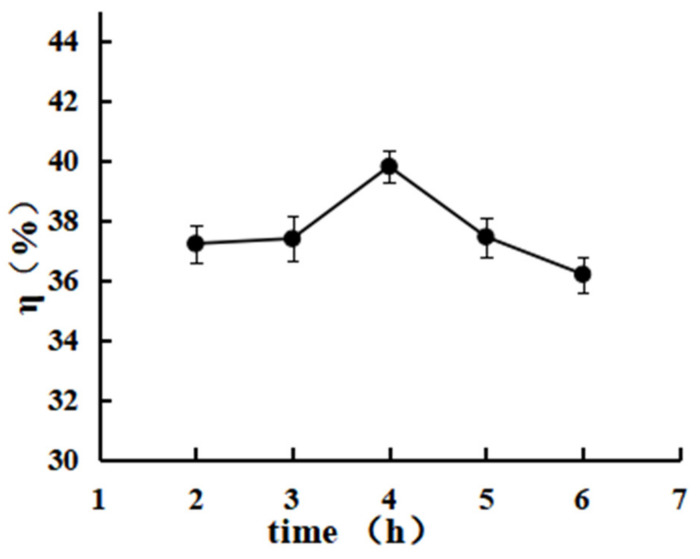
Effect of the reaction time on the grafting rate.

According to Figure 6, the η% of CTS-NF increased from 37.2 ± 1.21 to 39.8 ± 0.98 when the reaction time was extended from 2 to 4 h, while the η% decreased to 37.4 ± 1.92 and 36.2 ± 1.31, respectively, when the reaction time was further extended to 5 and 6 h. 

#### 3.3.3. Effect of the Reaction Temperature on the Grafting Rate

**Figure 7:**
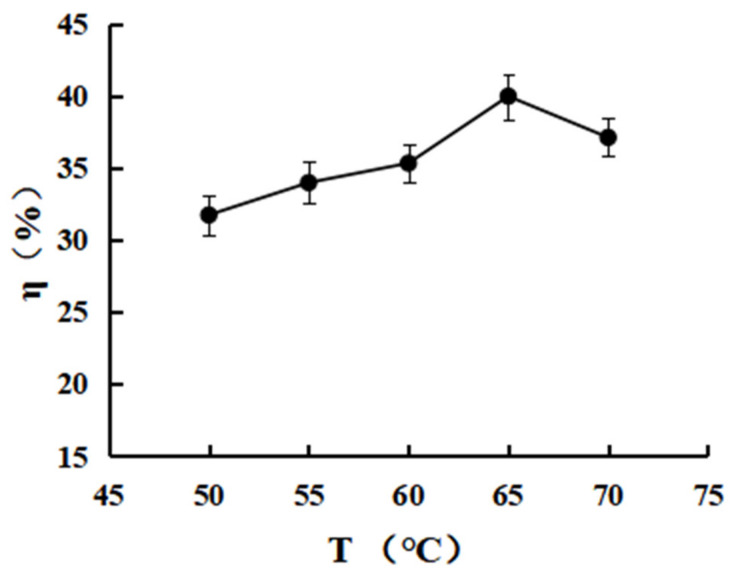
Effect of the reaction temperature (°C) on the grafting rate.

According to Figure 7, the η% of CTS-NF increased from 31.6 ± 2.12 to 40.0 ± 3.25 when T was increased from 50 to 65 °C, while the η% instead decreased to 37.1 ± 1.96 as T was increased further, which was probably because the dissolution rate of the reactants increased as the temperature increased at the initial stage of the reaction, allowing the reaction to reach the desired reaction entropy more rapidly, thereby accelerating the reaction rate. However, as the temperature continued to rise, the intermediates produced from NF activation with EDC were less stable and hydrolyzed, resulting in a decrease in the η%.

### 3.4. Response Surface Analysis

The Box–Behnken design was used, and the response surface analysis factors and levels used are shown in Table 1, the test results are shown in Table 2, and the regression quadratic model Equation (4) is given as:

Y = 45.50-1.25A − 0.45B + 0.20C − 0.035AB + 0.51AC − 0.076BC − 12.83A2 − 3.77B2 − 5.59C2(4)

The regression equation was subject to ANOVA and the results are presented in Table 3. The R^2^ of the secondary model equation was 0.9693 with *p* < 0.01, indicating that the model was highly significant. Additionally, the misfit term was 0.0838 (*p* > 0.05), which was not significant, indicating that the model equation was reliable and could be used to predict the η% of CTS-NF. The strength order of the three factors affecting grafting rate was: M_NF_/M_CTS_>T>t. At the significance level (*p* < 0.05), the single factor A (*p* = 0.0365) was significant, the secondary terms A2 (*p* < 0.0001), B2 (*p* = 0.0008) and C2 (*p* < 0.0001) exhibited high significance, while all other factors were not significant.

**Figure 8:**
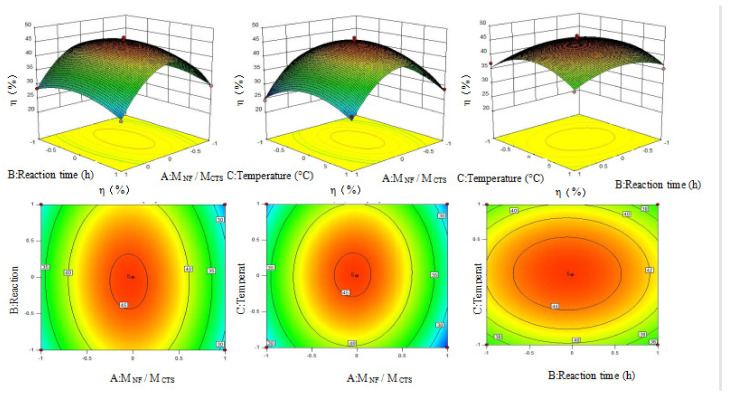
Three-dimensional effect surface plots of the dependent variable (η%) and three independent variables (M_NF_/M_CTS_, T and t).

According to the contour plots and three-dimensional (3D) effect surface plots of the effect of A and B, A and C, and B and C on the η% (Figure 8), the contour plots of A–B and A–C are clearly elliptic and the curvature of the 3D effect surface plots are clear. These indicate that the interaction of A–B and A–C has a clear impact on the η%. By contrast, the contour plots of B–C are inclined to be circular, and the curvature of the 3D effect surface plot is not as obvious as the former, which suggests that the interaction of B–C has little effect on the η%.

The optimal value was predicted using the response surface methodology and the results revealed that the η% was 45.5% when M_CTS_/M_NF_ was 5:3, T was 65 °C, and t was 4 h. The η% of CTS-NF was validated to be 45.8% by tests, which was close to the simulated value, demonstrating the goodness of the fit of the model.

### 3.5. Antibacterial Activity

The antimicrobial activity of CTS-NF was assessed by a broth dilution method against gram-negative *E. coli* ATCC 25922 and gram-positive *S. aureus* ATCC 29213. As shown in Table 4, the results indicate that CTS-NF exhibits a high activity againsgram-negative *E. coli* ATCC 25922 and gram-positive *S. aureus* ATCC 29213. The MIC values of CTS-NF are 0.3125 mg/mL against *E. coli* ATCC 25922, The MIC values of CTS-NF are 0.1562 mg/mL against *S. aureus* ATCC 29213. Compared with CTS, the MIC of the two bacteria was significantly reduced by CTS-NF. The antimicrobial activity of CTS has been well proven in past studies but the mechanism is yet to be discovered. The most acceptable interpretation is that the anionic cell surface of the microbes interacts with the cationic CTS, causing extensive cell surface alterations and damage. Norfloxacin inhibits the synthesis and replication of DNA by acting on the A subunit of bacterial DNA helicase, resulting in bacterial death. It is clear that grafting NF into CTS molecules can significantly improve the antibacterial activity of CTS-NF, thus making up for the low antibacterial activity of CTS.

### 3.6. Whole Blood Clotting and Red Blood Cell Aggregation

**Figure 9:**
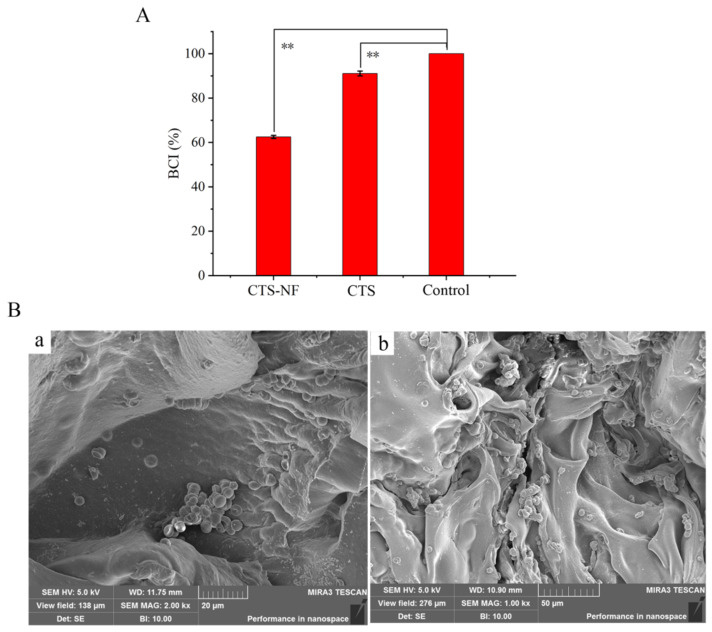
BCI of the samples (**A**); the SEM micrographs of the adsorption of red blood cells on the CTS-NF sponge surfaces (**B**), (**a**) the electron microscope of 2.00 kx; (**b**) the electron microscope of 1.00 kx). ** *p* < 0.01 indicates significant difference from the control group.

The BCI results of the samples are presented in Figure 9. The BCI of medical gauze was 91.1 ± 1.1% (Figure 9A), which was not significantly different from that of the blank group (*p* > 0.05). The BCI of CTS-NF was 62.5 ± 0.7%, which was significantly different from that of medical gauze (*p* < 0.01). The results revealed a significant coagulation effect of CTS-NF. Red blood cell adsorption tests were performed to better understand the coagulation-promoting mechanism of CTS-NF sponges, and the results of electron microscope observation are presented in Figure 9B. As observed, red blood cells were absorbed onto CTS-NF sponge in the form of clumps, because positively charged CTS can attract negatively charged red blood cells, and the hemostatic mechanism is not associated with the coagulation cascade [29]. Additionally, the high porosity of the CTS-NF sponge facilitated the aggregation of red blood cells on its surface, thus promoting the aggregation of red blood cells and accelerating the coagulation reaction.

### 3.7. Blood Plasma Coagulation

Among the four in vitro coagulation assays of prothrombin time (PT), activated partial thromboplastin time (APTT), thrombin time (TT) and (fibrinogen) FIB [30] are the most commonly used screening tests to reflect the exogenous and endogenous coagulation pathways, respectively, while TT is used to detect the contribution of prothrombin to thrombin production and FIB is used as a fibrinogen assay. 

As shown in Table 5, the PT, APTT, TT, and FIB values were statistically significantly reduced in the CTS-NF group compared to that of the blank control group (*p* < 0.05). These results revealed that CTS-NF may stimulate the production of a series of coagulation factors through prothrombin activation to produce thrombin [31], which act together through both, the exogenous coagulation and endogenous coagulation pathways, to accelerate the conversion of fibrinogen to insoluble fibrin in the blood, thus exhibiting a significant coagulation effect.

### 3.8. Animal Hemostasis Experiment

#### 3.8.1. Ear Artery Bleeding

**Figure 10:**
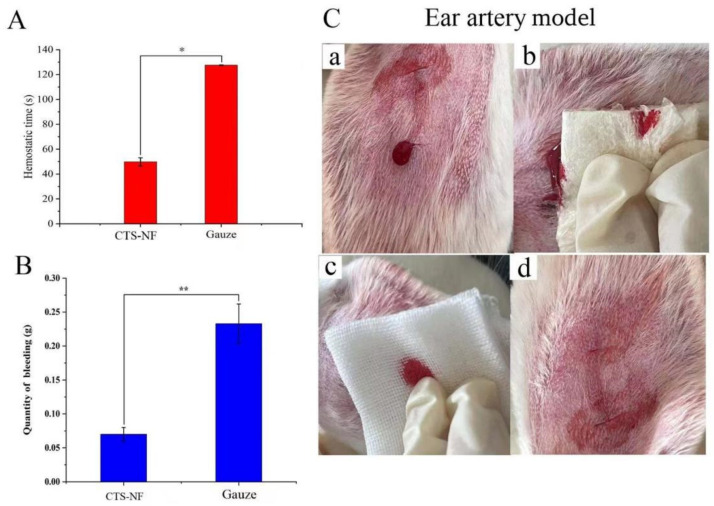
(**A**) The hemostasis time of the ear and (**B**) the amount of bleeding. (**C**) Ear artery hemostasis: bleeding (**a**), CTS-NF sponge hemostasis (**b**), medical gauze hemostasis (**c**), CTS-NF sponge wound (**d**). * *p* < 0.05, ** *p* < 0.01 indicates significant difference from the control group.

The results of the hemostasis tests at the ear artery in rabbits are presented in Figure 10A,B. During the hemostatic treatment, the medical gauze adhered poorly, whereas the CTS-NF sponge adhered well to the wound (Figure 10C). The hemostasis time in the medical gauze and CTS-NF groups was 127.55 ± 0.21 and 49.75 ± 3.32 s, respectively (Figure 10A), thus the hemostasis time in the NF group was statistically significant reduced by 156% compared with the medical gauze group (*p* < 0.01). By contrast, the amount of bleeding in the CTS-NF group of 0.07 ± 0.010 g was not significantly different compared with the gauze group of 0.233 ± 0.029 g (*p* < 0.01). In summary, the hemostatic time in the CTS-NF group was significantly less than that in the medical gauze group.

#### 3.8.2. Liver Bleeding

**Figure 11:**
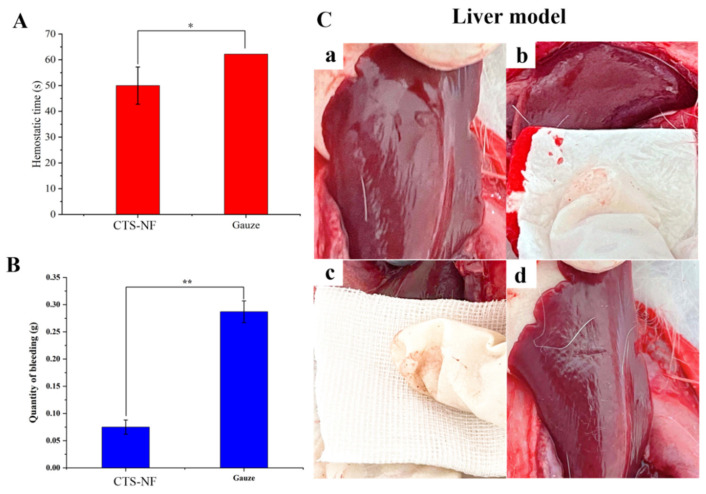
(**A**) The hemostasis time of the liver and (**B**) the amount of bleeding. (**C**) The liver model: Liver model bleeding (**a**), CTS-NF sponge hemostasis (**b**), medical gauze hemostasis (**c**), CTS-NF sponge wound (**d**). All data are expressed as the mean ± SD, *n* = 6). * *p* < 0.05, ** *p* < 0.01 indicates significant difference from the control group.

Figure 11C displays a New Zealand rabbit liver hemorrhage model, and the results of hemostatic tests on rabbit liver are presented in Figure 11A,B. As shown in Figure 11C, the CTS-NF sponge adhered well to the wound, whereas the medical gauze adhered poorly. The hemostasis time in the medical gauze group was 62.20 ± 2.13 s and that in the CTS-NF group was 50.00 ± 7.21 s (Figure 11A), which was statistically significantly 24.4% shorter compared to the medical gauze group (*p* < 0.05). The amount of blood absorbed in the medical gauze group was 0.287 ± 0.020 g compared with the CTS-NF group of 0.075 ± 0.013 g, which was statistically significantly different (*p* < 0.01). In conclusion, because of the good adhesion of CTS-NF to the trauma site, the hemostatic time in the CTS-NF group was significantly less than that in medical gauze group, with a good hemostatic effect on the liver.

The test results of hemostasis in vitro and in vivo indicated that due to a better hemostatic effect, the hemostasis time of the composite was significantly reduced compared with that of the medical gauze on the market. This was probably because of the high density of positive charge of CTS that can interact with the receptors containing cytosine residues on the surface of red blood cells, thus causing the red blood cells to coagulate on the material surface to form a thrombus [32,33]. Furthermore, CTS can also adsorb negatively charged plasma proteins and adhere to and activate platelets through electrostatic force, which allows platelets and other blood substances to gather on the surface to form a thrombus, thus playing a blood coagulation role [34,35]. Grafting NF can enhance the antibacterial properties of the sponge because it can effectively inhibit gram-positive cocci and gram-negative bacteria. Therefore, CTS-NF can enhance the antibacterial ability of sponge to resist bacterial infection during the hemostasis process [36].

## 4. Conclusions

On the basis of previous work, CTS-NF composite sponges with different grafting rate were prepared using a freeze-drying technique to obtain an excellent hemostatic material. The factors affecting the synthesis process were analyzed by single-factor tests, and the synthesis process was optimized using the response surface methodology. It was concluded that the optimal synthesis conditions were an M_CTS_/M_NF_ of 5:3, a T of 65 °C, and a t of 4 h, hereby achieving a maximum η% of 45.5% under these optimal reaction conditions. This study revealed that the CTS-NF composite sponge has strong inhibitory effects on both, *E. coli* and SA in the antibacterial tests. Additionally, the CTS-NF composite sponge exhibited good hemostatic activity in both, in vitro and in vivo hemostatic tests, but the specific mechanism of action requires further investigation.

## Figures and Tables

**Table 1:** Factors and levels of the response surface analysis.

Factor	Code	Standard		
		−1	0	1
M_CTS_/M_NF_	A	0.4	0.6	0.8
Reaction time (h)	B	3	4	5
Temperature (°C)	C	60	65	70

**Table 2:** Response surface experimental data results.

Run	A	B	C	η(%)
	M_CTS_/M_NF_	Reaction Time (h)	Temperature (°C)	
1	1	−1	0	28.6
2	1	1	0	26.2
3	−1	0	−1	27.3
4	0	0	0	45.4
5	0	0	0	45.5
6	0	0	0	45.5
7	1	0	−1	24.3
8	0	0	0	44.9
9	0	−1	−1	37.0
10	0	−1	1	35.5
11	0	1	−1	37.0
12	−1	−1	0	30.9
13	−1	1	0	29.9
14	0	0	0	44.5
15	1	0	1	27.8
16	−1	0	1	28.8
17	0	1	1	35.1

**Table 3:** Analysis of variance.

Source of Variance	Sum of Square	Degrees of Freedom	Mean Square	F Value	*p* Value
Model	972.61	9	108.07	57.16	<0.0001
A	12.58	1	12.58	6.66	0.0365
B	1.60	1	1.60	0.85	0.3883
C	0.33	1	0.33	0.18	0.6865
AB	0.50	1	0.50	0.26	0.6231
AC	1.02	1	1.02	0.54	0.4865
BC	0.023	1	0.023	0.012	0.9153
A^2^	693.50	1	693.50	366.83	<0.0001
B^2^	59.78	1	59.78	31.62	0.0008
C^2^	131.63	1	131.63	69.63	<0.0001
Residual	13.23	7	1.89		
Lack of fit	10.32	3	3.44	4.73	0.0838
Pure error	2.91	4	0.73		
Total deviation	985.84	16			
Coefficient of determination R^2^ = 0.9693					

**Table 4:** In vitro antimicrobial activity of CTS-NF.

Microorganism	MIC (mg/mL)		
	CTS-NF	CTS	NF
*E. coli* ATCC 25922	0.3125	2.5000	0.0008
*S. aureus* ATCC 29213	0.1562	1.2500	0.0003

**Table 5:** Measurement results of four coagulation indicators.

Sample	PT (s)	APTT (s)	TT (s)	FIB (g/L)
Control	21.32 ± 4.20	45.07 ± 10.71	24.67 ± 5.77	7.18 ± 1.45
CTS-NF	14.33 ± 2.37 *	33.08 ± 6.68 *	13.17 ± 3.91 *	3.07 ± 0.53 *

* *p* < 0.05, compared to the control group.

## Data Availability

The data presented in this study are contained within the article.

## References

[1] Jeschke M.G., van Baar M.E., Choudhry M.A., Chung K.K., Gibran N.S., Logsetty S. (2020). Burn injury. Nat. Rev. Dis. Primers.

[2] Chen D., Liu X., Qi Y., Ma X.B., Wang Y., Song H.Z., Zhao Y.L., Li W.J., Qin J.L. (2022). Poly (aspartic acid) based self-healing hydrogel with blood coagulation characteristic for rapid hemostasis and wound healing applications. Colloids. Surf. B.

[3] Cao C.Y., Yang N., Zhao Y., Yang D.P., Hu Y.L., Yang D.L., Song X.J., Wang W.J., Dong X.C. (2021). Biodegradable hydrogel with thermo-response and hemostatic effect for photothermal enhanced anti-infective therapy. Nano Today.

[4] Yu R., Zhang H., Guo B. (2022). Conductive biomaterials as bioactive wound dressing for wound healing and skin tissue engineering. Nano-Micro Lett..

[5] Zheng Y., Pan N., Liu Y., Ren X. (2021). Novel porous chitosan/N-halamine structure with efficient antibacterial and hemostatic properties. Carbohydr. Polym..

[6] Zhang Y., Dang Q., Liu C., Yan J., Cha D., Liang S., Li X., Fan B. (2017). Synthesis, characterization, and evaluation of poly(aminoethyl) modified chitosan and its hydrogel used as antibacterial wound dressing. Int. J. Biol. Macromol..

[7] Cheng Y., Wang J.W., Hu Z., Zhong S.Y., Huang N., Zhao Y.T., Tao Y., Liang Y.L. (2022). Preparation of norfloxacin-grafted chitosan antimicrobial sponge and its application in wound repair. Int. J. Biol. Macromol..

[8] Alkabli J. (2022). Progress in preparation of thiolated, crosslinked, and imino-chitosan derivatives targeting specific applications. Eur. Polym. J..

[9] Oryan A., Sahvieh S. (2017). Effectiveness of chitosan scaffold in skin, bone and cartilage healing. Int. J. Biol. Macromol..

[10] Azmana M., Mahmood S., Hilles A.R., Rahman A., Arifin M.A.B., Ahmed S. (2021). A review on chitosan and chitosan-based bionanocomposites: Promising material for combatting global issues and its applications. Int. J. Biolo. Macromol..

[11] Cao S.J., Xu G., Li Q.J., Zhang S.K., Yang Y.F., Chen J.D. (2022). Double crosslinking chitosan sponge with antibacterial and hemostatic properties for accelerating wound repair. Compos. Part B.

[12] Atashgahi M., Ghaemi B., Valizadeh A., Moshirib A., Nekoofar M.H., Amanid A. (2021). Epinephrine-entrapped chitosan nanoparticles covered by gelatin nanofibers: A bi-layer nano-biomaterial for rapid hemostasis. Int. J. Pharm..

[13] Lestari W., Yusry W.N.A.W., Haris M.S., Jaswir I., Idrus E. (2020). A glimpse on the function of chitosan as a dental hemostatic agent. Jpn. Dent. Sci. Rev..

[14] Lowrence R.C., Ramakrishnan A., Sundaramoorthy N.S. (2018). Norfloxacin salts of carboxylic acids curtail planktonic and biofilm mode of growth in ESKAPE pathogens. J. Appl. Microbiol..

[15] Ismail N.A., Amin K.A.M., Razali M.H. (2019). Antibacterial Study of Gellan Gum (GG) Film Incorporated Norfloxacin. J. Pure. Appl. Microbiol..

[16] Ismail N.A., Amin K., Razali M.H. (2018). Mechanical and Antibacterial Activities Study of Gellan Gum/Virgin Coconut Oil Film Embedded Norfloxacin. IOP Conf. Ser.: Mater. Sci. Eng..

[17] Dua K., Malipeddi V.R., Madan J., Gupta G., Chakravarthi S., Awasthi R., Kikuchi I.S., De T. (2018). Jesus Andreoli Pinto, Norfloxacin and metronidazole topical formulations for effective treatment of bacterial infections and burn wounds. Interv. Med. Appl. Sci..

[18] Khan M.A., Khan S., Kazi M., Alshehri S.M., Shahid M., Khan S.U., Hussain Z., Sohail M., Shafique M., Hamid H.A. (2021). Norfloxacin loaded lipid polymer hybrid nanoparticles for oral administration: Fabrication, characterization, in silico modelling and toxicity evaluation. Pharmaceutics.

[19] Ghafar A., Rozaik S., Akef A. (2019). Rifaximin plus norfloxacin versus norfloxacin alone in primary prophylaxis of spontaneous bacterial peritonitis in patients with variceal bleeding. Egypt. J. Int. Med..

[20] Goller S., Turner N.J. (2020). The antimicrobial effectiveness and cytotoxicity of the antibiotic-loaded chitosan: ECM scaffolds. Appl. Sci..

[21] Buyana B., Aderibigbe B.A., Ndinteh D.T., Fonkui Y.T., Kumar P. (2020). Alginate-pluronic topical gels loaded with thymol, norfloxacin and ZnO nanoparticles as potential wound dressings. J. Drug Delivery Sci. Technol..

[22] Koumentakou I., Terzopoulou Z., Michopoulou A., Kalafatakis I., Theodorakis K., Tzetzis D., Bikiaris D. (2020). Chitosan dressings containing inorganic additives and levofloxacin as potential wound care products with enhanced hemostatic properties. Int. J. Biol. Macromol..

[23] Yin M.L., Wang Y.F., Zhang Y., Ren X.H., Qiu Y.Y., Huang T.S. (2020). Novel quaternarized N-halamine chitosan and polyvinyl alcohol nanofibrous membranes as hemostatic materials with excellent antibacterial properties. Carbohydr. Polym..

[24] Wang X.Y., Li Q.L., Lu H.B., Liu Z., Wu Y.X., Mao J., Gong S.Q. (2022). Effects of the Combined Application of Trimethylated Chitosan and Carbodiimide on the Biostability and Antibacterial Activity of Dentin Collagen Matrix. Polymers.

[25] Yamada S., Yamamoto K., Ikeda T., Yanagiguchi K., Hayashi Y. (2014). Potency of fish collagen as a scaffold for regenerative medicine. Biomed. Res. Int..

[26] Wiegand I., Hilpert K., Hancock R.E.W. (2017). Agar and broth dilution methods to determine the minimal inhibitory concentration (MIC) of antimicrobial substances. Nat. Protoc..

[27] Catanzano O., D’Esposito V., Formisano P., Boatenget J.S., Quaglia F. (2018). Composite Alginate-Hyaluronan Sponges for the Delivery of Tranexamic Acid in Post-Extractive Alveolar Wounds. J. Pharmacol. Sci..

[28] Yan S.Z., Xie F.Y., Zhang S., Jiang L.Z., Qi B.K., Li Y. (2021). Effects of soybean protein isolate− polyphenol conjugate formation on the protein structure and emulsifying properties: Protein−polyphenol emulsification performance in the presence of chitosan. Colloids Surf. A.

[29] Hu Z., Zhang D.Y., Lu S.T., Li P.W., Li S.D. (2018). Chitosan-based composite materials for prospective hemostatic applications. Mar. Drugs.

[30] Ning Z., Liu Z., Song Z., Wang C., Liu Y., Gan J., Ma X., Lu A. (2017). Application of a strategy based on metabolomics guided promoting blood circulation bioactivity compounds screening of vinegar. Chem. Cent. J..

[31] Wang Y.W., Liu C.C., Cherng J.H., Lin C.S., Chang S.J., Hong Z.J., Liu C.C., Chiu Y.K., Hsu S.D., Chang H. (2019). Biological effects of chitosan-based dressing on hemostasis mechanism. Polymers.

[32] Mishra B., Hossain S., Mohanty S., Gupta M.K., Verma D. (2021). Fast acting hemostatic agent based on self-assembled hybrid nanofibers from chitosan and casein. Int. J. Biol. Macromol..

[33] Chen J.W., Ai J., Chen S.N., Xu Z.Y., Lin J.H., Liu H.Q., Chen Q.H. (2019). Synergistic enhancement of hemostatic performance of mesoporous silica by hydrocaffeic acid and chitosan. Int. J. Biol. Macromol..

[34] Fischer T.H., Thatte H.S., Nichols T.C., Bender-Neal D.E., Bellinger D.A., Vournakis J.N. (2005). Synergistic platelet integrin signaling and factor XII activation in poly-N-acetyl glucosamine fiber-mediated hemostasis. Biomaterials.

[35] Kou S.G., Peters L., Mucalo M. (2022). Chitosan: A review of molecular structure, bioactivities and interactions with the human body and micro-organisms. Carbohydr. Polym..

[36] Nor J.M.S., Khairul A.M.A. (2017). Composite Film of Chitosan Loaded Norfloxacin with Improved Flexibility and Antibacterial Activity for Wound Dressing Application. Orient. J. Chem..

